# Phenotype-based profiling of female sexual function in pregnancy: a cross-sectional study

**DOI:** 10.3389/fendo.2026.1874163

**Published:** 2026-06-17

**Authors:** Roberta Scairati, Nunzia Verde, Guendalina Del Vecchio, Francesco Castaldo, Chiara Graziadio, Nicola Tecce, Rosario Pivonello, Renata Simona Auriemma, Annamaria Colao

**Affiliations:** 1 Dipartimento di Medicina Clinica e Chirurgia, Sezione di Endocrinologia, Diabetologia, Andrologia e Nutrizione, Università Federico II di Napoli, Naples, Italy; 2 Dipartimento di Medicina Clinica e Chirurgia, Sezione di Endocrinologia, Diabetologia, Andrologia e Nutrizione, Unità di Andrologia e Medicina della Riproduzione, Sessualità e Affermazione di Genere, Università Federico II di Napoli, Naples, Italy; 3 UNESCO Chair for Health Education and Sustainable Development, Federico II University, Naples, Italy

**Keywords:** cluster analysis, female sexual function, gestational diabetes mellitus, pregnancy, sexual phenotypes

## Abstract

**Introduction:**

Sexual function during pregnancy is commonly described in terms of overall decline or sexual dysfunction prevalence, potentially overlooking heterogeneity across individual domains. We aimed to identify distinct sexual function phenotypes during pregnancy and to examine whether gestational diabetes mellitus (GDM) is associated with poorer sexual function or specific sexual profiles.

**Methods:**

In this cross-sectional observational study, 83 pregnant women undergoing routine GDM screening between weeks 24 and 28 of gestation completed the Female Sexual Function Index (FSFI). Clinical and obstetric data were collected. K-means cluster analysis on standardized FSFI domain scores identified multidomain phenotypes. Group comparisons and multivariable regression models were performed.

**Results:**

Sexual dysfunction was present in 51 of 83 women (61.4%). Three sexual phenotypes were identified: preserved sexual function (n=49), global sexual impairment (n=25), and desire-satisfaction dissociation (n=9). Pre-pregnancy weight differed across phenotypes (p=0.026). Hypertensive disorders of pregnancy, twin pregnancy, and conception by assisted reproductive technology (ART) were more frequent in the desire-satisfaction dissociation phenotype (p=0.024, p=0.042, and p=0.048, respectively). Older age (OR 1.15, 95% CI 1.01–1.32; p=0.041) and lower pre-pregnancy weight (OR 0.95, 95% CI 0.92–0.99; p=0.019) were independently associated with global sexual impairment. GDM was not associated with total FSFI score, sexual dysfunction prevalence, or phenotype distribution.

**Discussion:**

Female sexual function during pregnancy is heterogeneous and distributed across distinct multidomain phenotypes. Gestational diabetes mellitus did not explain this heterogeneity, whereas phenotype-based profiling identified distinctive sexual patterns. These findings support a more nuanced framework for studying sexuality in pregnancy beyond dichotomous definitions of dysfunction.

## 1 Introduction

Female sexual function during pregnancy is shaped by the constellation of physical, emotional, and relational changes. Across gestation, desire, arousal, lubrication, orgasm, satisfaction and pain may all be altered, although the extent and pattern of these changes are highly variable. Existing literature consistently shows that sexual function often worsens as pregnancy progresses, particularly in later gestation, but this decline is neither uniform nor domain-independent (1).

Most studies have approached sexuality in pregnancy as a global outcome, typically focusing on mean changes in sexual activity or on the prevalence of sexual dysfunction. Although this literature has generated important descriptive data, it may flatten a clinically heterogeneous experience. Individual domains of sexual function do not necessarily deteriorate in parallel, and sexual desire may remain partly dissociated from physiological sexual response. This variability appears to be shaped by multiple interacting factors, including age, physical discomfort, relational context, and pregnancy-related concerns, rather than by gestational status alone (2–5).

Whether metabolic complications of pregnancy contribute meaningfully to this heterogeneity remains unclear. Gestational diabetes mellitus (GDM) is a common pregnancy complication that introduces additional metabolic, psychological, and behavioral burdens (6, 7), yet its relationship with female sexuality remains poorly characterized. Most studies on sexual function in pregnancy have examined healthy or mixed obstetric populations, with limited attention to whether women with and without GDM may share or diverge in specific sexual function profiles. At the same time, factors frequently intertwined with GDM risk, such as age, adiposity, assisted reproductive technology (ART), and obstetric complexity, may independently influence sexual wellbeing and complicate simple dichotomous comparisons (8, 9).

A phenotype-based approach may therefore offer a more informative framework than the conventional classification of sexual dysfunction alone. Rather than assuming that pregnant women can be adequately described as having or not having sexual dysfunction, it may be more clinically meaningful to identify distinct multidomain profiles of sexual function and to examine their anthropometric, metabolic, and obstetric correlates.

In this cross-sectional study, we assessed female sexual function in a cohort of pregnant women undergoing routine screening for GDM in late second trimester. The primary aim was to identify and clinically characterize distinct Female Sexual Function Index (FSFI)-derived sexual phenotypes within the cohort. A secondary aim was to compare sexual function between women with GDM and normoglycemic pregnancies and to examine whether GDM status was associated with specific sexual profiles.

## 2 Methods

### 2.1 Study sample and procedure

This cross-sectional observational study was conducted at the Endocrinology, Diabetology, Andrology and Nutrition Unit, Department of Clinical Medicine and Surgery, “Federico II” University of Naples. Pregnant women were consecutively recruited during routine visits for GDM screening between the 24th and 28th weeks of gestation from January 2025 to February 2026. Eligible women were approached during the clinical evaluation, informed about the study, and invited to participate.

Inclusion criteria were age ≥18 years, singleton or twin pregnancy, gestational age between 24 and 28 weeks, ability to understand and complete the questionnaires in Italian, and provision of informed consent. Exclusion criteria were pregestational diabetes mellitus, inability to provide informed consent, and incomplete questionnaire data precluding analysis.

Before questionnaire administration, all participants received verbal information about the study and provided written informed consent.

Questionnaires were self-administered in a dedicated room, separately from the clinical evaluation. Clinical, anthropometric, and metabolic data were subsequently extracted from medical records and integrated with questionnaire-based information. This study was approved by the local ethics committee of “Federico II” University of Naples (protocol no. 304/2024; approval no. 00003276) and conducted in accordance with the Declaration of Helsinki.

### 2.2 Study measures

Female sexual function was assessed using the Female Sexual Function Index (FSFI), a validated multidimensional self-report instrument evaluating sexual function across six domains, including desire, arousal, lubrication, orgasm, satisfaction, and pain (10). The total FSFI score was calculated according to standard scoring procedures, with lower scores indicating poorer sexual function. Sexual dysfunction was defined using the conventional FSFI cut-off of ≤ 26.55 and the validated Italian version was used (11).

Adherence to the Mediterranean diet was assessed using the 14-item PREDIMED questionnaire, a validated tool measuring adherence to Mediterranean dietary habits through key nutritional components, including olive oil, fruit, vegetables, legumes, nuts, and fish intake. Based on the total score, adherence was categorized as poor, intermediate, or good according to the predefined study classification (12).

Maternal age, pre-pregnancy weight, pre-pregnancy body mass index (BMI), parity, previous miscarriage history, conception by ART, family history of diabetes, twin pregnancy, and hypertensive disorders of pregnancy were collected from routine clinical records, as well as smoking status, physical activity, reproductive history, and selected clinical and lifestyle information relevant to pregnancy.

Gestational weight change at the time of questionnaire administration was calculated in the subgroup of women for whom both pre-pregnancy and visit weight were available.

All women were referred for GDM screening and underwent metabolic evaluation according to routine clinical practice. Gestational diabetes diagnosis was established in accordance with current diagnostic criteria used in clinical care (13).

### 2.3 Statistical analysis

Given the exploratory, cross-sectional design of the study, no formal *a priori* sample size calculation was performed, and the sample size was determined by consecutive recruitment of eligible participants during the study period.

Statistical analyses were performed using SPSS for Mac OS, version 30.0 (IBM Corp., Armonk, NY, USA). Continuous variables are presented as mean ± standard deviation (SD) and, where appropriate, as median and range. Categorical variables are presented as counts and percentages.

Pearson’s correlation analysis was used to assess associations between continuous variables.

To explore heterogeneity in sexual function beyond the dichotomous FSFI classification, a k-means cluster analysis was performed using standardized FSFI domain scores in order to capture differences across individual dimensions of sexual function. Prior to clustering, FSFI domain scores were standardized to minimize the influence of scale variability across domains. The number of clusters was selected through combined evaluation of within-cluster sum of squares, silhouette analysis, and overall clinical interpretability of the identified profiles. The resulting clusters were considered exploratory and data-driven phenotypes rather than predefined biological categories.

Differences across sexual phenotypes were assessed using one-way analysis of variance (ANOVA) for continuous variables and the chi-square test for categorical variables. When normality assumptions were not met or when group sizes were small, non-parametric analyses were additionally performed using the Kruskal–Wallis test, followed, where appropriate, by pairwise comparisons with Bonferroni correction. To investigate independent correlates of sexual dysfunction, a binary logistic regression model was fitted with sexual dysfunction (yes/no) as the dependent variable and GDM status, maternal age, pre-pregnancy weight, conception by ART, and twin pregnancy as covariates. Comparisons between women with GDM and those with normoglycemic pregnancies were performed using the independent-samples t test for continuous variables and the chi-square test or Fisher’s exact test, as appropriate, for categorical variables.

An additional exploratory binary logistic regression analysis was performed using the global sexual impairment cluster versus the remaining clusters as the dependent variable, including maternal age, pre-pregnancy weight, GDM status, and ART conception as covariates. Results are reported as odds ratios (ORs) with 95% confidence intervals (CIs), where applicable, to facilitate interpretation beyond p-values alone. Additional exploratory analyses were performed to further characterize the desire–satisfaction dissociation cluster and the subgroup of women with GDM.

Statistical significance was set at p < 0.05.

## 3 Results

### 3.1 Study population and overall sexual function

A total of 83 pregnant women were included in this cross-sectional study, of whom 41 (49.4%) had GDM and 42 (50.6%) had normoglycemic pregnancies. Mean maternal age was 34.5 ± 4.4 years, and overall sexual dysfunction, according to the FSFI cut-off, was present in 51/83 women (61.4%), with a mean FSFI total score of 19.1 ± 11.2. Additional anthropometric and clinical characteristics of the study population are summarized in **Table 1**.

**Table 1:** Clinical, anthropometric, and sexual function characteristics of the study population.

Clinical variable	
Maternal age, years	34.5 ± 4.4 (34.0, 24–44)
Pre-pregnancy weight, kg	70.9 ± 20.5 (64.0, 43.0–132.0)
Pre-pregnancy BMI, kg/m^2^	26.7 ± 6.6 (25.4, 17.3–44.1)
GDM, n (%)	41/83 (49.4)
Gestational weight change at questionnaire, kg	4.1 ± 4.8 (4.0, −11.5 to 18.5)
First pregnancy, n (%)	45/81 (55.6)
Previous miscarriage history, n (%)	24/82 (29.3)
ART conception, n (%)	14/82 (17.1)
Family history of diabetes, n (%)	43/82 (52.4)
Hypertensive disorders of pregnancy, n (%)	12/83 (14.5)
Twin pregnancy, n (%)	4/79 (5.1)
Regular physical activity, n (%)	21/83 (25.3)
Smoking status	
Never smoker, n (%)	55/83 (66.3)
Former smoker, n (%)	22/83 (26.5)
Current smoker, n (%)	6/83 (7.2)
Mediterranean diet adherence (PREDIMED)	
Poor adherence, n (%)	21/83 (25.3)
Intermediate adherence, n (%)	53/83 (63.9)
Good adherence, n (%)	9/83 (10.8)
FSFI total score	19.1 ± 11.2 (23.2, 2.0–35.0)
FSFI desire	3.6 ± 1.2 (3.6, 1.2–6.0)
FSFI arousal	2.8 ± 2.3 (3.6, 0.0–6.0)
FSFI lubrication	3.0 ± 2.5 (4.2, 0.0–6.0)
FSFI orgasm	3.0 ± 2.5 (4.0, 0.0–6.0)
FSFI satisfaction	3.9 ± 1.8 (4.4, 0.8–6.0)
FSFI pain	2.8 ± 2.5 (3.6, 0.0–6.0)
Sexual dysfunction (yes/no), n (%)	51/83 (61.4)

GDM, gestational diabetes mellitus; ART, Assisted reproductive technology; FSFI, Female sexual function index.

Correlation analyses in the overall cohort revealed selective associations between maternal characteristics and sexual function. Maternal age was modestly and inversely correlated with sexual desire (r = −0.225, p = 0.041) and satisfaction (r = −0.212, p = 0.050). By contrast, pre-pregnancy weight was positively correlated with arousal (r = 0.297, p = 0.006), lubrication (r = 0.324, p = 0.003), orgasm (r = 0.290, p = 0.008), satisfaction (r = 0.232, p = 0.035), pain (r = 0.249, p = 0.023), and the overall FSFI score (r = 0.297, p = 0.006).

Pre-pregnancy BMI showed positive correlations with arousal (r = 0.216, p = 0.050), lubrication (r = 0.260, p = 0.018), and orgasm (r = 0.222, p = 0.044), whereas gestational weight change was not significantly associated with FSFI measures.

### 3.2 FSFI-derived sexual phenotypes and their clinical correlates

**Figure 1:**
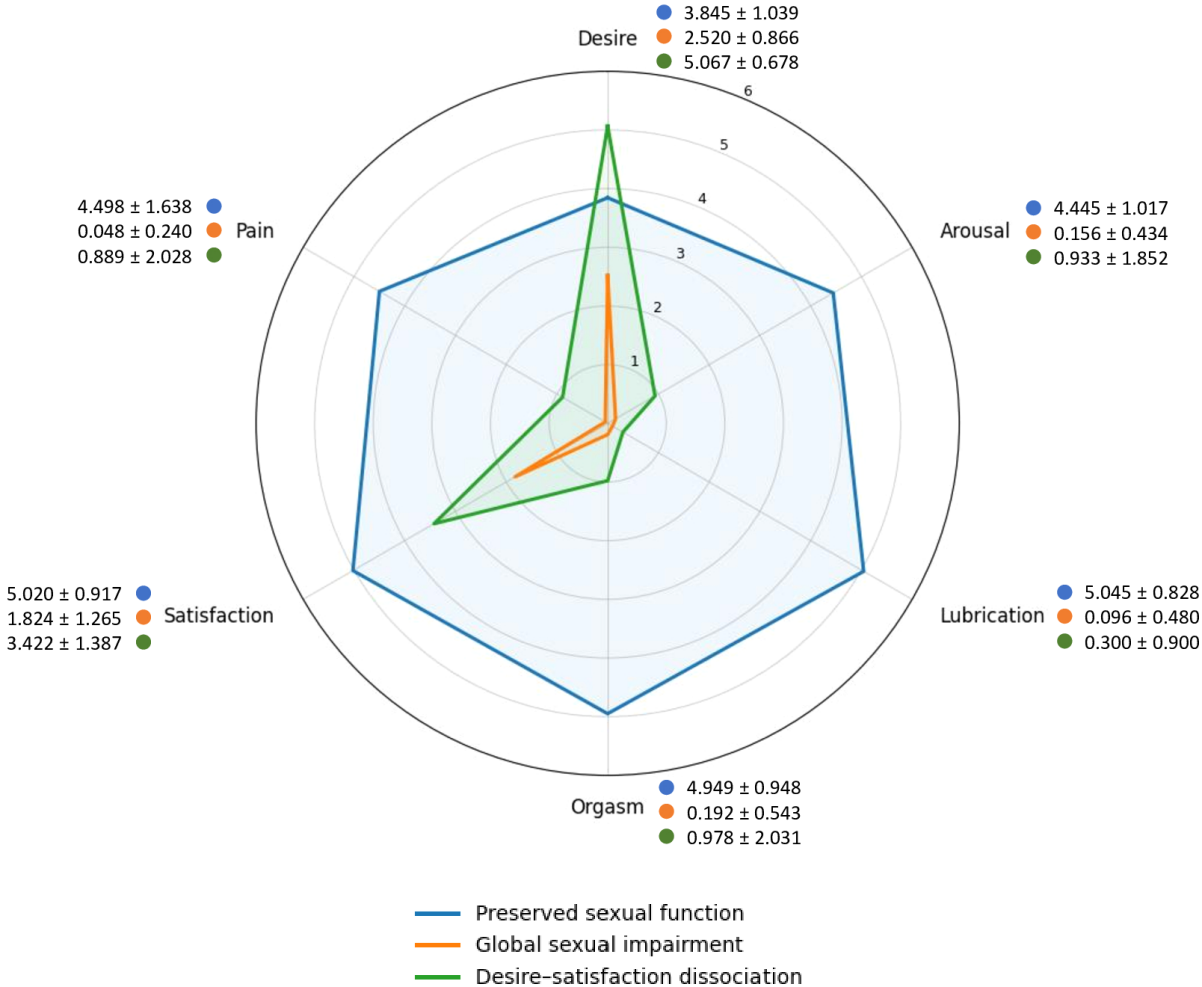
Mean raw FSFI domain scores across the three sexual phenotypes in pregnancy. The preserved sexual function cluster showed relatively high scores across all domains (blue line), the global sexual impairment cluster showed uniformly low scores (orange line), and the desire–satisfaction dissociation cluster was characterized by relatively preserved desire in the context of lower scores in the remaining domains (green line). Mean ± SD values for each FSFI domain are shown alongside each domain label, with color-coded markers corresponding to the respective cluster profiles. **Figure 1** was generated with BioRender.com.

To further explore heterogeneity in sexual function, a k-means cluster analysis based on standardized FSFI domain scores identified three distinct sexual phenotypes within the cohort. The largest cluster (n = 49) was characterized by relatively preserved scores across all domains and was defined as the preserved sexual function cluster. A second cluster (n = 25) showed uniformly low scores across all FSFI domains, representing a global sexual impairment cluster. The third cluster (n = 9) displayed a distinct pattern characterized by high desire scores in the presence of low arousal, lubrication, orgasm, and pain scores, with intermediate satisfaction. This phenotype was defined as a desire–satisfaction dissociation cluster, suggesting a decoupling between sexual desire and physiological sexual response. Standardized cluster centers and raw FSFI domain scores are reported in **Tables 2** and **3**, respectively, and the distribution of these three profiles is illustrated in **Figure 1**.

**Table 2:** Final cluster centers based on standardized FSFI domain scores (z-scores).

FSFI domain	Cluster 1 Preserved sexual function	Cluster 2 Global sexual impairment	Cluster 3 Desire-satisfaction dissociation
*Desire*	0.21602	-0.85758	1.20605
*Arousal*	0.73886	-1.15572	-0.81234
*Lubrication*	0.79162	-1.16219	-1.08165
*Orgasm*	0.75262	-1.16866	-0.85130
*Satisfaction*	0.63142	-1.14513	-0.25684
*Pain*	0.68343	-1.07279	-0.74092
Cluster size, n	49	25	9

**Table 3:** Raw FSFI domain scores across sexual phenotypes.

FSFI domain	Cluster 1 preserved sexual function mean ± SD	Cluster 2 global sexual impairment mean ± SD	Cluster 3 desire-satisfaction dissociation mean ± SD
*Desire*	3.845 ± 1.039	2.520 ± 0.866	5.067 ± 0.678
*Arousal*	4.445 ± 1.017	0.156 ± 0.434	0.933 ± 1.852
*Lubrication*	5.045 ± 0.828	0.096 ± 0.480	0.300 ± 0.900
*Orgasm*	4.949 ± 0.948	0.192 ± 0.543	0.978 ± 2.031
*Satisfaction*	5.020 ± 0.917	1.824 ± 1.265	3.422 ± 1.387
*Pain*	4.498 ± 1.638	0.048 ± 0.240	0.889 ± 2.028
Cluster size, n	49	25	9

Clinical and anthropometric characteristics according to sexual function phenotypes are summarized in **Table 4**.

**Table 4:** Clinical, anthropometric, and obstetric characteristics across FSFI-derived sexual phenotypes.

Variable	Overall	Cluster 1	Cluster 2	Cluster 3	*P value*
Maternal age, years	34.54 ± 4.38	34.20 ± 4.34	35.68 ± 3.98	33.22 ± 5.47	0.250
Pre-pregnancy BMI, kg/m^2^	26.68 ± 6.63	28.27 ± 7.41	24.41 ± 4.35	24.34 ± 5.20	**0.030**
Pre-pregnancy weight, kg	70.92 ± 20.51	76.75 ± 23.50	62.42 ± 11.20	62.78 ± 10.69	**0.020**
GDM, n/N (%)	41/83 (49.4)	27/49 (55.1)	10/25 (40.0)	4/9 (44.4)	0.447
First pregnancy, n/N (%)	45/81 (55.6)	25/48 (52.1)	16/25 (64.0)	4/8 (50.0)	0.590
Hypertensive disorders of pregnancy, n/N (%)	12/83 (14.5)	9/49 (18.4)	0/25 (0.0)	3/9 (33.3)	**0.024**
Twin pregnancy, n/N (%)	4/79 (5.1)	1/46 (2.2)	1/24 (4.2)	2/9 (22.2)	**0.042**
ART conception, n/N (%)	14/82 (17.1)	8/49 (16.3)	2/24 (8.3)	4/9 (44.4)	**0.048**
Family history of diabetes, n/N (%)	43/82 (52.4)	29/48 (60.4)	10/25 (40.0)	4/9 (44.4)	0.222
Physical activity, n/N (%)	21/83 (25.3)	15/49 (30.6)	5/25 (20.0)	1/9 (11.1)	0.357
Previous miscarriages, n/N (%)	24/82 (29.3)	15/48 (31.3)	5/25 (20.0)	4/9 (44.4)	0.345
**Smoking status**					0.692
Never smoker, n/N (%)	55/83 (66.3)	32/49 (65.3)	16/25 (64.0)	7/9 (77.8)	
Former smoker, n/N (%)	22/83 (26.5)	12/49 (24.5)	8/25 (32.0)	2/9 (22.2)	
Current smoker, n/N (%)	6/83 (7.2)	5/49 (10.2)	1/25 (4.0)	0/9 (0.0)	
**Mediterranean diet adherence (PREDIMED)**					0.825
Poor adherence, n/N (%)	21/83 (25.3)	12/49 (24.5)	7/25 (28.0)	2/9 (22.2)	
Intermediate adherence, n/N (%)	53/83 (63.9)	32/49 (65.3)	16/25 (64.0)	5/9 (55.6)	
Good adherence, n/N (%)	9/83 (10.8)	5/49 (10.2)	2/25 (8.0)	2/9 (22.2)	

GDM, gestational diabetes mellitus; ART, Assisted reproductive technology. P values were calculated using one-way ANOVA for continuous variables and Pearson’s chi-square test for categorical variables.

Bold values indicate statistically significant p-values (p < 0.05).

No significant difference was observed in the distribution of GDM across the three clusters.

Pre-pregnancy BMI and weight differed across sexual phenotypes, with higher values observed in the preserved sexual function cluster (**Table 4**). However, after non-parametric testing, only pre-pregnancy weight remained significantly associated with phenotype distribution (p = 0.026).

No significant associations were observed between sexual phenotypes and Mediterranean diet adherence, parity, previous miscarriage history, family history of diabetes, smoking status, or physical activity.

However, selected obstetric characteristics differed across phenotypes. Hypertensive disorders of pregnancy were more frequent in the desire-satisfaction dissociation cluster compared with others (p = 0.024). Twin pregnancies and conception by ART were also more frequent in this group (p = 0.042 and p = 0.048, respectively).

An exploratory multivariable logistic regression analysis identified maternal age and pre-pregnancy weight as independent correlates of the global sexual impairment cluster. Older age was associated with higher odds of belonging to this cluster (OR 1.15, 95% CI 1.01–1.32, p = 0.041), whereas higher pre-pregnancy weight was associated with lower odds (OR 0.95, 95% CI 0.92–0.99, p = 0.019).

By contrast, neither GDM status nor conception by ART was independently associated with this cluster.

Additional exploratory analyses further characterized the desire–satisfaction dissociation cluster by comparing women in this group with the remaining pregnant women.

Conception by ART was more frequent in the desire–satisfaction dissociation cluster and showed an approximately fivefold increased association with this profile (OR 5.04, 95% CI 1.15–22.02). Twin pregnancy was also more frequent in the desire–satisfaction dissociation cluster (2/9, 22.2% vs 2/70, 2.9%, p = 0.042), although this association did not remain significant in exact testing. No significant associations were observed with GDM status, anthropometric measures, or lifestyle variables.

### 3.3 Sexual function in women with GDM and normoglycemic pregnancies

Mean total FSFI score was similar in women with GDM (20.35 ± 10.16) and in those with normoglycemic pregnancies (17.93 ± 12.14) (p = 0.326). Likewise, the prevalence of sexual dysfunction did not differ significantly between the two groups (27/41, 65.9%, vs 24/42, 57.1%; p = 0.415). No significant differences were observed between women with GDM and normoglycemic pregnancies across individual FSFI domains.

In multivariable logistic regression analysis, the association between sexual dysfunction and clinically relevant maternal characteristics was further explored by including GDM status, maternal age, pre-pregnancy weight, conception by assisted reproductive technology (ART), and twin pregnancy in the same model. No variable emerged as an independent correlate of sexual dysfunction, and the overall model showed limited explanatory ability.

Within the GDM subgroup, maternal age was inversely associated with the FSFI satisfaction domain (r = −0.325, p = 0.038). Pre-pregnancy weight was positively correlated with arousal (r = 0.382, p = 0.014), lubrication (r = 0.353, p = 0.024), pain (r = 0.341, p = 0.029) and total FSFI score (r = 0.348, p = 0.026).

## 4 Discussion

Sexual function in pregnancy is biologically and clinically heterogeneous. In this cohort of women undergoing routine screening for GDM in late second trimester, more than 60% met the conventional FSFI threshold for sexual dysfunction, confirming that impairment of sexual wellbeing is common at this stage of gestation. This finding is broadly consistent with previous reports, although prevalence estimates vary substantially across cohorts, gestational timing, and assessment strategies (4). More importantly, rather than pointing to a single trajectory of impaired sexual function, the identification of distinct FSFI-derived phenotypes supports the view that sexuality in pregnancy is multidimensional, heterogeneous, and shaped by factors extending beyond gestational status alone.

In the overall cohort, maternal age showed modest inverse associations with sexual desire and satisfaction, consistent with reports suggesting that older age may be associated with greater sexual distress during pregnancy (14–16). A plausible explanation is that women experiencing pregnancy later in reproductive life may adopt a more cautious sexual attitude or perceive pregnancy as more fragile.

By contrast, the positive association between pre-pregnancy weight and multiple FSFI domains was less expected and should be interpreted cautiously. One possible explanation is that women with higher baseline body weight may experience body changes as less abrupt or less disruptive to body confidence than women starting pregnancy from a leaner baseline. Alternatively, pre-pregnancy weight may reflect unmeasured psychosocial or relational variables not captured in the present dataset. These findings should not be interpreted as evidence of a beneficial effect of greater body weight per se, but rather as further evidence that the relationship between anthropometry and sexual function during pregnancy is likely multifactorial.

The most original aspect of the present study lies in the phenotype-based characterization of sexual function during pregnancy. Rather than relying exclusively on the dichotomous definition of sexual dysfunction, FSFI domain scores were used to explore multidomain patterns within the cohort. This approach delineated three profiles: preserved sexual function, global sexual impairment, and a smaller desire–satisfaction dissociation pattern. These profiles should not be interpreted as fixed biological entities, but rather as exploratory configurations capturing the heterogeneity of sexual experience in pregnancy. Women with comparable overall FSFI scores may still differ substantially across individual sexual domains, including desire, arousal, orgasm, satisfaction, and pain.

The global sexual impairment cluster was characterized by uniformly low scores across all FSFI domains and, in multivariable analysis, by older maternal age and lower pre-pregnancy weight. This finding is consistent with the correlations observed in the whole cohort and suggests that age and pre-pregnancy anthropometry may shape broader sexual profiles during pregnancy rather than isolated FSFI domains alone.

The desire–satisfaction dissociation cluster was smaller but clinically intriguing. Women in this group showed relatively high desire despite low arousal, lubrication, orgasm, and pain scores, with intermediate satisfaction.

Prior literature supports the view that sexual domains do not necessarily deteriorate in parallel during pregnancy, and that desire may follow a trajectory distinct from arousal, intercourse-related comfort, and orgasmic response, with some studies reporting a second-trimester peak (1), while others suggest that desire may remain relatively preserved compared with other domains in specific subgroups of women (17). Interestingly, similar dissociations between preserved sexual desire and impaired sexual functioning have also been described in women with Polycystic Ovary Syndrome (PCOS)/Polyendocrine Metabolic Ovarian Syndrome (PMOS), suggesting that affective and motivational components of sexuality may remain partially preserved despite impaired physiological sexual response (18).

In our cohort, hypertensive disorders of pregnancy, ART conception, and twin pregnancy were more frequently observed in the desire-satisfaction dissociation cluster, although these findings should be interpreted cautiously given the limited sample size and exploratory nature of the analysis. One possible hypothesis is that pregnancies characterized by greater medical complexity may differentially influence individual dimensions of sexuality, potentially contributing to a dissociation between motivational and physiological sexual components.

In such settings, increased bodily vigilance, perceived pregnancy fragility, and concerns for maternal or fetal wellbeing may disproportionately affect somatic aspects of sexuality, while affective and motivational components may remain relatively preserved.

However, the present study was not designed to investigate the psychological, relational, or neurobiological mechanisms underlying this pattern, and no causal inference can be drawn. Therefore, these observations should be considered hypothesis-generating and warrant targeted investigation in larger cohorts.

The second aim of this study was to determine whether GDM was associated with poorer sexual function or with a distinct sexual profile. No significant association was observed in our cohort. Women with GDM had FSFI total scores and prevalence of sexual dysfunction comparable to those of women with normoglycemic pregnancies, and GDM was not independently associated with sexual dysfunction in multivariable analysis. Although GDM is defined by abnormal glucose regulation, it also reflects a broader endocrine and metabolic adaptation occurring during pregnancy (19a, 20b). However, its impact on sexual function may be insufficient to shape a distinct sexual phenotype, particularly within the relatively limited gestational window in which GDM develops and is clinically managed. In addition, many women rapidly enter structured care pathways after diagnosis, including dietary counseling, glucose monitoring, and clinical follow-up, which may mitigate metabolic and psychosocial burden. This interpretation is also consistent with the subgroup analyses in women with GDM, where maternal age and pre-pregnancy weight retained selective associations with individual FSFI domains, but no signal suggested that glycemic status alone reorganized sexual profiles in a consistent way.

No association emerged between Mediterranean diet adherence and either sexual phenotypes or overall sexual dysfunction. Although this variable was included because of its potential relevance to metabolic and behavioral health during pregnancy, these findings suggest that dietary pattern was not a major determinant within this cohort.

This study has several limitations. First, its cross-sectional design does not allow temporal or causal inference. Second, the cohort was relatively small, particularly for the desire–satisfaction dissociation cluster, limiting the robustness of subgroup analyses. Third, sexual function was assessed through self-administered questionnaires, and several relevant psychosocial variables, including partner-related factors, sexual distress, anxiety, depressive symptoms, body image, and pre-pregnancy sexual function were not systematically assessed. Finally, the phenotype-based approach is exploratory by design and requires external validation in independent cohorts.

These limitations are balanced by several strengths. The study was conducted in a clinically relevant window of pregnancy, included both women with GDM and normoglycemic pregnancies assessed within the same care setting, integrated metabolic and obstetric data, and moved beyond conventional dichotomous classifications of sexual dysfunction of clinically relevant heterogeneity that would not have been captured by total FSFI scores alone.

In conclusion, sexual dysfunction was common in this cohort of pregnant women, but sexual function was distributed across distinct multidomain profiles rather than along a single gradient of impairment. Gestational diabetes status alone did not account for this heterogeneity. By contrast, maternal age, pre-pregnancy anthropometry, and selected obstetric characteristics appeared more informative in shaping sexual profiles, particularly the global impairment and desire–satisfaction dissociation patterns. These findings support a more nuanced view of sexuality in pregnancy and provide a basis for future studies aimed at validating phenotype-based approaches in larger and more deeply characterized populations.

## Data Availability

The raw data supporting the conclusions of this article will be made available by the authors, without undue reservation.
